# Antioxidant Defense Systems in Plants: Mechanisms, Regulation, and Biotechnological Strategies for Enhanced Oxidative Stress Tolerance

**DOI:** 10.3390/life15081293

**Published:** 2025-08-14

**Authors:** Faustina Barbara Cannea, Alessandra Padiglia

**Affiliations:** Department of Life and Environmental Sciences (DiSVA), Biomedical Section, University of Cagliari, 09042 Monserrato, Italy; faustinab.cannea@unica.it

**Keywords:** oxidative stress, ROS, antioxidant defense, redox signaling, omics, CRISPR/Cas systems, synthetic biology, crop resilience

## Abstract

Plants must contend with oxidative stress, a paradoxical phenomenon in which reactive oxygen species (ROS) can cause cellular damage while also serving as key signaling molecules. Environmental stressors, such as drought, salinity, and temperature extremes, promote ROS accumulation, affecting plant growth and productivity. To maintain redox homeostasis, plants rely on antioxidant systems comprising enzymatic defenses, such as superoxide dismutase, catalase, and ascorbate peroxidase, and non-enzymatic molecules, including ascorbate, glutathione, flavonoids, and emerging compounds such as proline and nano-silicon. This review provides an integrated overview of antioxidant responses and their modulation through recent biotechnological advances, emphasizing the role of emerging technologies in advancing our understanding of redox regulation and translating molecular insights into stress-resilient phenotypes. Omics approaches have enabled the identification of redox-related genes, while genome editing tools, particularly those based on clustered regularly interspaced short palindromic repeats (CRISPR) and CRISPR-associated (Cas) proteins, offer opportunities for precise functional manipulation. Artificial intelligence and systems biology are accelerating the discovery of regulatory modules and enabling predictive modeling of antioxidant networks. We also highlight the contribution of synthetic biology to the development of stress-responsive gene circuits and address current regulatory and ethical considerations. Overall, this review aims to provide a comprehensive perspective on molecular, biochemical, and technological strategies to enhance oxidative stress tolerance in plants, thereby contributing to sustainable agriculture and food security in a changing climate.

## 1. Introduction

Reactive oxygen species (ROS) in plants including superoxide anion (O_2_^−^), hydrogen peroxide (H_2_O_2_), hydroxyl radicals (•OH), and singlet oxygen (^1^O_2_), are reactive byproducts of oxygen metabolism [1,2]. In addition, the reaction between superoxide and nitric oxide (NO) generates peroxynitrite (ONOO^−^), a highly reactive molecule that bridges ROS and reactive nitrogen species (RNS) signaling and contributes to redox-dependent cellular reprogramming [3,4]. While traditionally regarded as harmful molecules produced during aerobic respiration and photosynthesis, ROS are now recognized as dual-function agents in plant biology [5]. When tightly controlled, these oxidative molecules serve as key signaling intermediates in processes such as growth, development, stomatal function, and stress response, acting in concert with hormonal pathways and transcriptional networks [6]. However, when the accumulation of oxidants surpasses the neutralizing capacity of the antioxidant system, oxidative stress occurs, leading to cellular damage affecting lipids, proteins, and nucleic acids. This ultimately disrupts vital physiological functions and compromises development. The generation of ROS is intrinsic to aerobic metabolism and closely linked to photosynthesis, respiration, and β-oxidation [7]. Even in the absence of stress, low levels of reactive species are continuously produced through electron transport and enzymatic activity in compartments such as chloroplasts, mitochondria, peroxisomes, and the apoplast. Under favorable conditions, including adequate light, temperature, nutrients, and water, plants maintain ROS levels within physiological thresholds through a tightly regulated antioxidant network that includes the constitutive activity of enzymatic scavengers such as SOD, CAT, and APX, as well as non-enzymatic compounds like ascorbate and glutathione. In contrast, environmental stressors such as drought, salinity, temperature extremes, high light, nutrient imbalance, or heavy metal toxicity disturb redox homeostasis, resulting in an overaccumulation of oxidants and oxidative damage [8]. However, when ROS production exceeds the plant’s antioxidant capacity, the defense system becomes overwhelmed, and cellular damage becomes unavoidable. In chloroplasts, ROS generation is primarily linked to photosynthesis. When photosystem II (PSII) becomes oversaturated with excess light energy, ^1^O_2_ and O_2_^−^ are formed, leading to oxidative damage to the thylakoid membranes and photosynthetic pigments and compromising photosynthetic efficiency [9,10]. Similarly, mitochondria produce ROS through electron leakage from the respiratory chain, particularly at complexes I and III. Mitochondrial oxidants often act as signals for programmed cell death, a mechanism that is crucial in eliminating damaged cells and maintaining tissue function under prolonged stress. The biological effects of ROS are highly context dependent and regulated by their concentration, their subcellular localization, and the timing of their production [11]. At moderate levels, ROS, especially H_2_O_2_ due to its relative stability and membrane permeability, act as key signaling molecules, regulating processes such as stomatal movement, root growth, pollen tube elongation, senescence, and systemic stress responses. These signals are mediated by complex networks involving mitogen-activated protein kinase (MAPK) cascades; calcium signaling; and redox-sensitive transcription factors (TFs), such as WRKY (tryptophan–arginine–lysine–tyrosine), NAC (NAM, ATAF, and CUC), basic leucine zipper (bZIP), and APETALA2/ethylene-responsive factor (AP2/ERF). Crosstalk with phytohormones such as abscisic acid (ABA), salicylic acid (SA), jasmonic acid (JA), and ethylene further integrates ROS signaling into plant stress responses [12,13,14,15,16]. Conversely, excessive ROS levels can overwhelm the antioxidant system, causing extensive damage through lipid peroxidation, protein oxidation, DNA fragmentation, and enzyme inactivation, ultimately compromising plant growth, reproduction, and survival. Emerging evidence highlights the protective roles of novel antioxidant compounds such as proline, taurine, and nano-silicon. These molecules contribute to stress tolerance by functioning as osmoprotectants, direct ROS scavengers, or enhancers of antioxidant gene expression. For example, proline is particularly effective in terms of neutralizing •OH and stabilizing proteins and membranes under stress. Spatiotemporal ROS patterns, including oscillations and systemic ROS waves, play pivotal roles in stress memory and systemic acquired acclimation, enabling plants to mount faster and more robust responses to recurring stress episodes [17,18,19,20]. This spatial and temporal integration of oxidative signals contributes to the establishment of a primed physiological state This phenomenon, known as stress memory, allows plants to respond more quickly and effectively to repeated stress episodes. This dual role of reactive species, as both signaling agents and damaging oxidants, requires precise regulation of the antioxidant network. The next section explores the enzymatic and non-enzymatic elements that coordinate this regulation under stress conditions.

## 2. Antioxidant Defense Systems in Plants

Maintaining redox balance under fluctuating environmental conditions requires the precise coordination of antioxidant defenses. These defenses include both enzymatic and non-enzymatic components, which are compartmentalized within the cell and dynamically regulated. The following sections provide an overview of the main biochemical functions of antioxidant defenses, their compartmentalization within cellular organelles, and the transcriptional, epigenetic, and post-translational mechanisms regulating their activity in response to oxidative cues.

### 2.1. Enzymatic and Non-Enzymatic Antioxidants

Plants rely on a highly coordinated antioxidant system to manage ROS generated under both normal metabolic conditions and environmental stress. This system comprises two interdependent components: enzymatic antioxidants, which catalyze the removal of ROS through defined biochemical pathways, and non-enzymatic antioxidants, which act as direct scavengers or signaling modulators. In this section, we provide a comprehensive overview of these antioxidant elements, highlighting their mechanisms, subcellular localization, and roles in plant stress adaptation.

#### 2.1.1. Enzymatic Antioxidants

The enzymatic antioxidant system represents the first line of defense. Superoxide dismutases (SODs) catalyze the dismutation of O_2_^−^ into hydrogen peroxide (H_2_O_2_), which is less reactive but still harmful. This H_2_O_2_ is subsequently decomposed into water and oxygen by catalase (CAT), which is primarily located in peroxisomes, and by ascorbate peroxidase (APX), which exists in multiple isoforms across chloroplasts (stromal and thylakoid-bound), mitochondria, cytosol, and peroxisomes. While CAT operates efficiently at high H_2_O_2_ concentrations, APX functions under lower levels, playing a crucial role in fine-tuned detoxification during moderate oxidative stress [21,22,23].

These enzymes are integral in the ascorbate–glutathione (AsA–GSH) cycle, which is crucial in maintaining redox homeostasis. In this cycle, APX uses ascorbate to reduce H_2_O_2_ to water, producing monodehydroascorbate (MDHA). MDHA is either directly recycled to ascorbate by monodehydroascorbate reductase (MDHAR) using NADPH, or it can disproportionate into ascorbate and dehydroascorbate (DHA). The latter is subsequently reduced to ascorbate by dehydroascorbate reductase (DHAR) using glutathione (GSH). Glutathione reductase (GR) then reduces oxidized glutathione (GSSG) back to its active form (GSH) in an NADPH-dependent reaction. This enzymatic network not only eliminates H_2_O_2_ but also maintains high intracellular GSH/GSSG and ascorbate/DHA ratios, critical redox indicators during stress responses [24].

Additional enzymatic antioxidants include glutathione peroxidases (GPXs), which reduce H_2_O_2_ and lipid hydroperoxides using GSH, and peroxiredoxins (PRXs), which detoxify peroxides through electron donation by thioredoxins. These enzymes play dual roles in antioxidant defense and redox signaling and are differentially localized in organelles such as chloroplasts and mitochondria [25].

#### 2.1.2. Non-Enzymatic Antioxidants

Complementing enzymatic defenses, plants also rely on a diverse array of non-enzymatic antioxidants. These include ascorbate, glutathione, tocopherols, carotenoids, polyphenols, flavonoids, and proline [26,27]. These molecules act both as direct scavengers of oxidants and as modulators of redox-sensitive signaling pathways. In particular, they can chelate redox-active transition metals such as Fe^2+^ and Cu^+^, thereby preventing •OH formation via Fenton chemistry [28].

These compounds employ diverse biochemical strategies to neutralize oxidative damage. Ascorbate acts as an electron donor in a two-step redox mechanism, sequentially converting to MDHA and then to DHA, thereby scavenging H_2_O_2_, O_2_^−^, and •OH. Glutathione contributes through the thiol group of its cysteine residue, forming oxidized glutathione (GSSG) or participating in protein glutathionylation, a reversible modification that shields redox-sensitive cysteines from irreversible oxidation. Tocopherols terminate lipid peroxidation chains by donating a hydrogen atom to lipid peroxyl radicals, generating a relatively stable tocopheroxyl radical that can be regenerated by ascorbate [29]. Carotenoids quench ^1^O_2_ through physical energy transfer to their conjugated double-bond system, releasing energy as heat, and also neutralize peroxyl radicals (ROO•) [30]. Flavonoids and other phenolic compounds act via electron or hydrogen atom donation and chelation of Fe^2+^ and Cu^+^ ions. Their antioxidant potency is strongly influenced by hydroxylation patterns, particularly at the 3′ and 4′ positions of the B ring.

For instance, carotenoids (e.g., β-carotene, lutein) and tocopherols are embedded in cellular membranes, where they prevent oxidative damage by quenching ^1^O_2_ and limiting lipid peroxidation under high light stress. Flavonoids and other phenolics not only scavenge reactive species but also regulate redox-sensitive signaling cascades by interacting with kinases and TFs, thereby influencing stress-responsive gene expression [31]. Ascorbate also functions as both a direct antioxidant and a cofactor in enzymatic detoxification reactions, while glutathione participates in the AsA–GSH cycle and in protein glutathionylation, contributing to fine-tuned redox regulation under oxidative stress.

**Figure 1:**
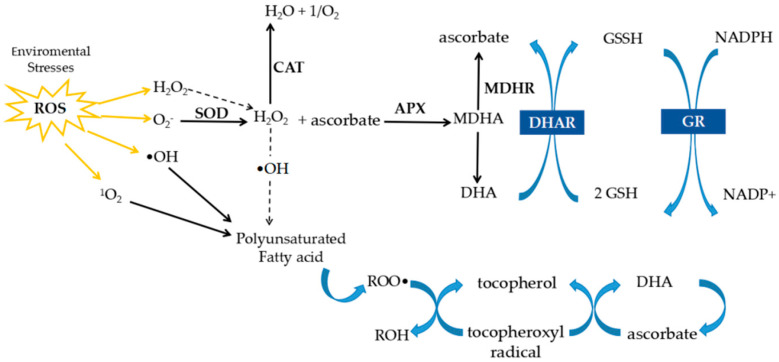
Schematic representation of the ascorbate–glutathione cycle. Ascorbate scavenges ROS, leading to DHA formation. DHA is reduced back to ascorbate by DHAR using GSH as electron donor, while GR regenerates GSH from GSSG, sustaining redox homeostasis. Arrows indicate the direction of the reactions in the ascorbate–GSH cycle.

A schematic representation of the AsA–GSH cycle is shown in Figure 1. In this pathway, ascorbate plays a key role in scavenging ROS, including H_2_O_2_, and is oxidized to DHA. DHA is then recycled back to ascorbate by DHAR using GSH as a reducing agent, while GR restores GSH from its oxidized form (GSSG), thereby maintaining cellular redox balance. This cycle not only removes ROS but also regulates the GSH/GSSG and AsA/DHA ratios, which are critical markers of redox homeostasis.

Together, enzymatic and non-enzymatic systems form a dynamic and spatially organized defense network that is essential for regulating ROS-mediated signaling and maintaining redox equilibrium during stress responses.

### 2.2. Subcellular Localization and Dynamic Regulation

A distinctive feature of plant antioxidant defenses is their subcellular compartmentalization, with ROS being produced and neutralized in organelles depending on metabolic flux and environmental stimuli (Table 1).

Chloroplasts, due to their intense photosynthetic activity, are major sites of oxidative species formation and require robust, localized detoxification systems involving SOD isoforms and the AsA–GSH cycle. Mitochondria generate ROS mainly at complexes I and III, where electron leakage is modulated by redox buffering mechanisms that depend on Mn-SOD and GSH availability [11]. Peroxisomes, conversely, function as H_2_O_2_-generating hubs during photorespiration and fatty acid oxidation, relying on CAT and additional components of the AsA–GSH cycle for rapid detoxification [32]. Beyond this compartment-specific activity, recent evidence suggests that antioxidant capacity in each organelle is dynamically regulated, both transcriptionally and post-translationally, in response to fluctuating oxidative cues. This regulation allows localized redox balance while maintaining systemic antioxidant coordination.

In addition to compartment-specific detoxification, antioxidant capacity is modulated by small signaling molecules such as NO and melatonin, which can act upstream of gene expression or modulate post-translational events. For instance, exogenous melatonin increases the activities of SOD, CAT, and APX in various crops, while NO influences the redox state of transcriptional regulators and enzymes, contributing to senescence delay and stress tolerance [33].

Moreover, emerging biostimulants, including taurine, nano-silicon, and plant-derived peptides, enhance oxidative stress resilience by mimicking endogenous redox signals or enhancing the expression of antioxidant genes. Nano-silicon, for example, boosts CAT and APX activities and reduces lipid peroxidation under heat stress [34,35]. Recent studies using transcriptomic profiling and redox-sensitive biosensors have shown that antioxidant responses are orchestrated with spatiotemporal precision. Local detoxification events, such as those occurring in guard cells or root tips, are coordinated with systemic responses via ROS waves, propagated by respiratory burst oxidase homolog D (RBOHD)-mediated H_2_O_2_ production, and Ca^2+^-dependent signaling cascades involving cyclic nucleotide-gated channels (CNGCs) and calcium-dependent protein kinases (CDPKs). These processes contribute to systemic acquired acclimation, priming distal tissues to respond more efficiently to future stress episodes through a phenomenon known as stress memory [36].

While previously considered a passive legacy of stress, stress memory is now understood as an active reprogramming process that involves transient but functionally significant biochemical, transcriptional, and epigenetic adjustments. This redox-based priming enables plants to optimize the balance between growth and defense during recurrent stress events. These long-distance signaling processes not only coordinate antioxidant activity but also contribute to the establishment of stress memory through redox-based priming of distal tissues [37].

### 2.3. Transcriptional and Epigenetic Regulation of Antioxidant Defenses

**Figure 2:**
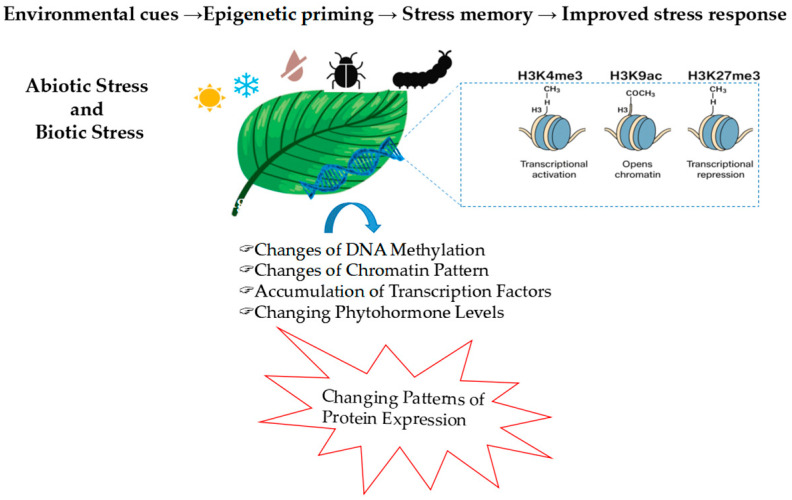
Epigenetic regulation of plant responses to abiotic (e.g., heat, drought, cold) and biotic (e.g., pathogens, herbivores) stress. These environmental cues induce chromatin modifications, including histone methylation (H3K4me3 for transcriptional activation, H3K27me3 for repression), histone acetylation (H3K9ac for chromatin relaxation), and DNA methylation changes. Together, these epigenetic marks modulate the transcription of stress-responsive genes, enabling redox regulation, priming, and the establishment of stress memory.

At the molecular level, the transcriptional regulation of antioxidant genes is mediated by networks involving the TFs from the AP2/ERF, WRKY, NAC, and bZIP families [38]. These factors act as central hubs that integrate oxidative signals with hormonal pathways, including ABA, SA, and JA, to modulate the expression of detoxifying enzymes such as SOD, APX, and CAT. H_2_O_2_, in particular, influences TFs activity through two main mechanisms. First, it can oxidize redox-sensitive cysteine residues, inducing conformational changes that affect DNA-binding affinity, subcellular localization, and protein–protein interactions [39]. Second, it can activate MAPK cascades that trigger post-translational modifications (PTMs) such as phosphorylation, S-nitrosylation, and glutathionylation, further modulating TF stability and function. For instance, WRKY TFs in *Arabidopsis thaliana* are regulated by cysteine oxidation, which alters their affinity for W-box promoter elements [40]. ABI5, a bZIP TF involved in ABA signaling, is phosphorylated by MAPKs in response to oxidative stress, enhancing its nuclear accumulation and transcriptional activity. Once activated, these TFs bind to specific cis-regulatory elements in the promoters of antioxidant genes. Through this mechanism, redox signals influence not only detoxification but also broader physiological processes such as stomatal dynamics, root meristem function, and leaf senescence. These responses are further modulated by hormonal crosstalk and Ca^2+^/MAPK signaling [41,42]. High-throughput genomics and transcriptomics have revealed redox-inducible promoter elements that underlie genotypic differences in antioxidant capacity. Stress-tolerant cultivars frequently show enhanced basal or inducible expression of detoxification genes. Moreover, transgenic plants overexpressing antioxidant genes or enzymes involved in GSH biosynthesis display improved tolerance to drought, salinity, and heavy metal stress. Beyond transcriptional control, epigenetic mechanisms play a critical role in regulating antioxidant defenses under oxidative stress [43]. Histone modifications, such as H3K4me3 (associated with transcriptional activation), H3K9ac (histone acetylation that opens chromatin), and H3K27me3 (a repressive mark), modulate chromatin accessibility at ROS-responsive loci. In *Arabidopsis*, the accumulation of H3K4me3 at the promoters of APX1 (ascorbate peroxidase 1) and ZAT10 (zinc finger transcription factor 10) has been associated with transcriptional priming and stress memory formation. Conversely, H3K27me3 is often removed during ROS accumulation, enabling derepression of antioxidant-related genes [44]. In rice, drought stress induces H3K9ac at the promoters of SOD and CAT, correlating with their activation [44]. DNA methylation contributes to long-term transcriptional regulation. In maize, hypomethylation at redox-responsive loci has been associated with increased antioxidant gene expression and improved redox resilience [45]. In parallel, chromatin remodeling complexes, such as SWI/SNF-type ATPases, adjust chromatin accessibility to support transcriptional reprogramming during oxidative stress [46]. Finally, epigenetic control converges with PTMs. Phosphorylation of WRKY and bZIP TFs influences their nuclear localization and DNA-binding activity, while S-nitrosylation regulates APX1, GSH1, and other redox-responsive proteins [47]. Together, these transcriptional, epigenetic, and post-translational layers of regulation enable plants to respond flexibly and effectively to oxidative challenges while maintaining developmental balance. A growing body of evidence suggests that oxidative stress not only triggers transient gene expression, but also establishes a physiological imprint known as stress memory (Figure 2). This primed state enables plants to respond more rapidly and effectively to recurring stress by modulating antioxidant defenses at multiple levels. Redox cues, particularly H_2_O_2_, initiate this process by inducing systemic signals such as ROS waves and Ca^2+^-dependent cascades, which activate key transcriptional modules and hormonal pathways (e.g., ABA, SA). At the molecular level, stress memory involves sustained or more rapidly inducible expression of genes such as APX1, ZAT10, and WRKY53, alongside persistent chromatin modifications. Histone marks like H3K4me3 and H3K9ac remain enriched at antioxidant loci after stress, facilitating rapid transcriptional reactivation. Conversely, the removal of repressive marks like H3K27me3 contributes to gene derepression upon repeated exposure [43,44,45]. DNA hypomethylation and chromatin remodeling further support this transcriptional persistence. Together, these molecular mechanisms underpin a form of redox-based priming that enhances stress resilience while minimizing unnecessary energy expenditure. Understanding the transcriptional and epigenetic basis of stress memory offers promising avenues for priming strategies and the development of crops with improved oxidative stress tolerance. These regulatory layers establish a dynamic molecular framework for redox adaptation, further explored in the next section on genetic and hormonal control of antioxidant responses.

## 3. Multilayered Regulation of Antioxidant Gene Expression

Antioxidant responses in plants are orchestrated through a complex regulatory hierarchy involving transcriptional networks, redox-sensitive signaling, phytohormones, and epigenetic mechanisms. Moderate levels of ROS, particularly H_2_O_2_, act as signaling intermediates that modulate the expression of both enzymatic and non-enzymatic antioxidants. This section explores how TFs, hormonal pathways, small molecules, and chromatin-based mechanisms interact to fine-tune antioxidant gene expression under stress conditions.

### 3.1. Transcriptional Control and Redox-Sensitive TFs

H_2_O_2_ is a key mediator of redox signaling due to its stability and diffusibility across membranes. It modifies the activity of TFs either through direct oxidation of cysteine residues or by triggering kinase cascades. The resulting transcriptional response includes the upregulation of genes encoding major antioxidant enzymes such as SOD, APX, CAT, and GR. Several transcription factor families act as integrators of redox signals. WRKYs, particularly WRKY33, are activated via MAPK cascades and regulate APX and CAT expression. Members of the NAC family, such as ANAC017 and OsNAC6, connect mitochondrial and ER-derived ROS signals with nuclear transcriptional responses [48,49,50]. The bZIP family includes bZIP28 and bZIP60, which mediate oxidative stress adaptation in the endoplasmic reticulum. AP2/ERF TFs, especially the DREB subgroup, integrate ROS signals with drought and salinity responses, while MYB proteins are involved in both flavonoid biosynthesis and the detoxification of ROS [51].

Many of these TFs are redox-sensitive and undergo post-translational modifications (PTMs), such as disulfide bond formation, S-nitrosylation, or phosphorylation. Thioredoxins and glutaredoxins modulate TF redox states, linking ROS levels to transcriptional control. In parallel, ROS waves trigger MAPK cascades that amplify oxidative signals and phosphorylate TFs, enhancing their activity and nuclear localization [52,53]. These kinase pathways also integrate ROS signaling with Ca^2+^-responsive modules, including CAMTA TFs, contributing to systemic transcriptional responses [54].

### 3.2. Hormonal and Epigenetic Modulation of Redox-Responsive Genes

Phytohormones reinforce redox-mediated transcriptional programs through TF activation and promoter binding. ABA induces antioxidant genes via ABA-responsive elements (ABREs) and TFs like ABI5. SA regulates redox signaling through the NPR1–TGA complex, while JA and ET signals are integrated through AP2/ERF TFs such as ERF1 [55,56,57].

In addition to hormonal signaling, emerging small molecules enhance antioxidant transcription. Melatonin upregulates WRKY and NAC TFs and boosts APX and CAT activity, particularly under drought and salinity stress. NO regulates the AsA–GSH cycle via S-nitrosylation of redox enzymes and TFs, increasing redox buffering capacity [58]. Biostimulants such as nano-silicon, taurine, and humic acids act through priming and epigenetic remodeling, enhancing the speed and intensity of antioxidant gene activation. Epigenetic mechanisms are also essential for the transcriptional plasticity of redox genes. Histone acetylation (e.g., H3 and H4 hyperacetylation) and DNA demethylation at promoters of APX1 and CAT2 have been linked to stress-induced gene activation. Moreover, microRNAs (miRNAs) offer a layer of post-transcriptional regulation. For example, miR398 modulates SOD levels by targeting its mRNA for degradation. Under mild stress, miR398 is upregulated to prevent unnecessary antioxidant activity, while severe stress leads to its downregulation, allowing SOD accumulation. Other miRNAs, such as miR395, influence glutathione biosynthesis by regulating sulfate assimilation pathways [59,60].

This multilayered regulatory network allows plants to fine-tune antioxidant defenses with high contextual precision, ensuring both responsiveness and metabolic efficiency.

### 3.3. Organelle Signaling and Biotechnological Applications

Redox signals originating in chloroplasts and mitochondria play an active role in retrograde signaling pathways that regulate the expression of nuclear antioxidant genes. In chloroplasts, retrograde signaling is mediated by multiple redox-sensitive pathways. Changes in the redox state of the plastoquinone pool, resulting from imbalances in electron transport during photosynthesis, activate GENOMES UNCOUPLED 1 (GUN1), a central integrator of plastid-derived signals that modulate nuclear gene expression [61]. In parallel, the accumulation of ^1^O_2_ under high light stress activates EXECUTER 1 (EX1)-dependent signaling, which leads to transcriptional reprogramming of nuclear antioxidant and stress-related genes [62]. Mitochondrial oxidative stress, on the other hand, promotes the cleavage and nuclear translocation of NAC-domain TFs such as ANAC017 in *Arabidopsis*, reinforcing nuclear redox homeostasis. Biotechnological strategies increasingly exploit this regulatory knowledge to enhance antioxidant capacity and stress tolerance [63,64]. Transgenic plants overexpressing TFs such as *AtWRKY30*, *OsNAC10*, and *SlERF84* exhibit elevated levels of SOD, CAT, and glutathione peroxidase, resulting in improved resilience to drought and salinity. Genome editing tools, particularly CRISPR/Cas9, enable precise modification of cis-regulatory elements to drive stress-inducible gene expression [65].

Synthetic promoters that combine ROS-, ABA-, and pathogen-responsive elements are also being developed to achieve conditional expression of stress-protective genes while minimizing energy and fitness costs. Furthermore, the integration of transcriptomic, proteomic, and epigenomic data continues to reveal central redox hubs and regulatory networks, such as the NPR1–TGA complex and redox-sensitive alternative splicing events, that shape antioxidant responses under complex environmental conditions [66].

## 4. Omics Insights into Plant Responses to Redox Stress

In recent years, omics technologies have revolutionized our ability to dissect the complexity of plant responses to oxidative stress. Traditional physiological and biochemical assessments of antioxidant activity have been increasingly complemented—and often surpassed—by high-throughput platforms such as genomics, transcriptomics, proteomics, and metabolomics. These approaches enable the systematic identification of key regulatory genes, redox-related pathways, and stress-responsive signaling networks, thereby providing a comprehensive understanding of the antioxidant defense system [67].

### 4.1. Genomics and Genome-Wide Approaches

Genomic studies help elucidate antioxidant defense mechanisms, enabling the identification of the genes encoding enzymatic and regulatory components. Whole-genome sequencing (WGS) and comparative genomics have revealed the conserved gene families encoding key antioxidant enzymes, including SODs, CATs, APXs, GPXs, and thioredoxins [68,69]. Although structurally conserved, these genes often exhibit divergent expression profiles under stress, reflecting regulatory variation across species and cultivars. Genome-wide association studies (GWAS) and quantitative trait locus (QTL) mapping have linked natural variation in stress tolerance to specific genomic loci [70,71]. For example, GWAS in *Arabidopsis* and foxtail millet have identified loci associated with glutathione metabolism and ascorbate cycling, highlighting genes such as glutathione S-transferase U19 (GSTU19), glutathione synthase 1 (GSH1), and vitamin C defective 2 (VTC2) as candidates for involvement in redox buffering capacity. In major crops, including rice, wheat, and tomatoes, next-generation sequencing (NGS) has facilitated the construction of pan-genomes that encompass both core antioxidant genes and cultivar-specific stress-responsive loci, emphasizing the genomic plasticity underlying antioxidant responses. For instance, a GWAS conducted on rice (a C_3_ species) identified salt tolerance-associated loci within vitamin E biosynthesis pathways, while a parallel study on maize (a C_4_ species) revealed polymorphisms in genes related to glutathione metabolism, suggesting divergent antioxidant strategies between species with distinct photosynthetic systems [72,73,74,75].

### 4.2. Transcriptomics and Gene Expression Profiling

Among the available omics platforms, transcriptomics remains one of the most widely applied tools in terms of exploring plant oxidative stress responses. RNA sequencing (RNA-seq) quantitatively profiles gene expression under diverse environmental conditions, enabling the identification of differentially expressed genes (DEGs). Key antioxidant-related DEGs, such as APX1, CAT2, SOD1, DHAR1, and the TFs WRKY53, NAC42, and ZAT12, are consistently upregulated in response to abiotic stressors such as drought, salinity, and high light [76,77].

Beyond DEG analysis, transcriptomic data network analysis (WGCNA), which facilitates the identification of central regulatory nodes, or hub genes. For example, in cold-stressed oil palm, TFs of the C-repeat binding factor (CBF) family have been shown to co-regulate antioxidant genes and function as master regulators. In winter oilseed rape (*Brassica napus*), transcriptome analysis of cold-tolerant and cold-sensitive cultivars revealed over 4000 DEGs and more than 130 differentially accumulated proteins (DAPs), particularly in ROS scavenging and hormone-related pathways [78]. In contrast, in rice under cold stress, WGCNA identified DREB TFs as master regulators of co-expressed antioxidant gene modules, indicating species-specific transcriptomic strategies [79]. Transcriptomics has also uncovered widespread alternative splicing events that modulate antioxidant enzyme diversity. Stress-induced splicing variants of APX6 and GST have been identified, with potential functional implications for protein activity during oxidative bursts. Additionally, cold-responsive transcriptomic networks in winter wheat reinforce the centrality of redox-responsive gene expression in stress tolerance [80].

### 4.3. Integration with Functional Validation

Transcriptome-based discoveries are increasingly integrated with functional validation techniques to confirm gene function and relevance in vivo. Common validation strategies include virus-induced gene silencing (VIGS) for the transient suppression of gene expression, the overexpression of candidate genes in model plants such as *Arabidopsis thaliana* or *Nicotiana benthamiana*, and CRISPR/Cas9-mediated genome editing to precisely knock out or modulate gene activity [81,82,83,84,85]. Recent applications include the targeted editing of GSH1 and NPR1 in rice and tomatoes, revealing gene-dosage-dependent effects on oxidative stress tolerance and redox homeostasis. Different functional validation approaches have been applied depending on species and gene targets. For example, VIGS has been used in *Arabidopsis* and *Nicotiana* to transiently knock down WRKY53 and other redox-responsive TFs during senescence and oxidative bursts. In contrast, CRISPR/Cas9 editing of GSH1 and NPR1 in rice has revealed gene dosage-dependent effects on oxidative stress resilience and redox buffering capacity [86]. Overexpression studies have further demonstrated that enhanced expression of GST, APX, SOD, and CAT genes can improve tolerance to abiotic stress by modulating redox equilibrium. For instance, knockout and overexpression of APX isoforms in tomato confirmed dose-dependent effects on redox balance. A transcriptome-guided CRISPR strategy in tomato validated ROS-regulatory modules identified by RNA-seq, while high-throughput gene function analysis via CRISPR in crops is emerging as a scalable tool for antioxidant network engineering [87,88].

### 4.4. Proteomics and Post-Transcriptional Regulation

Proteomics complements transcriptomics by providing direct insights into protein abundance, isoform diversity, stability, and post-translational modifications (PTMs). Techniques such as liquid chromatography–tandem mass spectrometry (LC-MS/MS), isobaric tags for relative and absolute quantitation (iTRAQ), and two-dimensional difference gel electrophoresis (2D-DIGE) allow the characterization of dynamic changes in redox-related proteins under stress condition [89,90].

Proteomic analyses in crops such as rice and maize exposed to oxidative stress have identified crucial players in redox homeostasis, including heat shock proteins (HSPs), peroxiredoxins, and glutaredoxins, some of which are not transcriptionally regulated, underscoring the importance of post-transcriptional control. Comparative proteomic studies have shown that targeted iTRAQ analyses in oilseed crops can detect lipid peroxidation-related proteins under oxidative stress, while label-free proteomics in maize highlights heat shock proteins and glutaredoxins that are not transcriptionally regulated, emphasizing the role of post-translational regulation. Post-translational modifications such as S-nitrosylation and phosphorylation modulate the activity, subcellular localization, and interaction of antioxidant enzymes [91]. For example, redox-regulated PTMs of APX and CAT have been shown to impact enzymatic efficiency under stress [92]. Advances in proteomics technologies have enabled deeper exploration of such modifications in complex plant systems. Furthermore, proteomic approaches have begun to elucidate stress memory-related changes in redox proteins, showing that PTM profiles shift following repeated exposure to environmental challenges [93].

### 4.5. Metabolomics and Redox Pathway Mapping

Metabolomics provides a comprehensive snapshot of the biochemical state of plant cells and reflects real-time responses to oxidative stress and antioxidant activity. Targeted and untargeted metabolomics enable the quantification of key metabolites, such as ascorbate, glutathione, malondialdehyde (MDA), and redox-active secondary compounds, including flavonoids. Analytical platforms such as gas chromatography–mass spectrometry (GC-MS), liquid chromatography–mass spectrometry (LC-MS), and nuclear magnetic resonance (NMR) have revealed that oxidative stress conditions induce the accumulation of γ-aminobutyric acid (GABA), polyamines, and phenylpropanoids such as quercetin and ferulic acid, which significantly contribute to antioxidant capacity [94,95]. The stress-induced accumulation of intermediates in the AsA–GSH cycle, including DHA and GSSG, highlights the pivotal role of redox cycling in plant stress adaptation. In cadmium-exposed rice, GC–MS analyses revealed increases in GABA and phenylpropanoid compounds, while LC–MS confirmed the accumulation of redox metabolites such as DHA and GSSG, illustrating the complementary power of targeted and untargeted metabolomics platforms [96,97]. Comparative metabolomic studies also demonstrated elevated levels of quercetin and ferulic acid under abiotic stress.

### 4.6. Multi-Omics Integration and Systems Biology

Integrated omics approaches provide a comprehensive and systems-level perspective on redox regulation [98]. Computational tools such as MapMan, OmicsNet, and Cytoscape facilitate the visualization and interpretation of gene–protein–metabolite interaction networks, revealing coordinated regulatory hubs [99,100,101]. In oil palm, integrated transcriptomic and metabolomic analyses performed under cold stress revealed tight coordination between CBF TFs, APX isoforms, and phenylpropanoid biosynthetic enzymes, indicating multilayered redox regulation. In tomatoes, WRKY–flavonoid regulatory modules have been identified as key ROS mitigation hubs, integrating gene expression with secondary metabolism. In *Populus* hybrids, an integrated transcriptomic and metabolomic study using OnPLS modeling compared wild-type and antisense SOD lines, revealing systemic differences in glycolysis and the oxidative pentose phosphate pathway. Similarly, in tomato, Systems-level insights into WRKY–flavonoid regulatory modules in tomato under carbon nanomaterial exposure [102,103].

Moreover, machine learning (ML) and artificial intelligence (AI) models are increasingly being applied to prioritize candidate antioxidant genes based on co-expression patterns, transcriptional responsiveness, and regulatory centrality. These approaches accelerate gene discovery, breeding strategies, and genome-editing pipelines [104,105].

However, the implementation of AI and ML in plant sciences still faces important challenges, including the need for high-quality and diverse datasets, potential algorithmic biases, and the limited interpretability of complex models [106].

### 4.7. From Omics to Application: Breeding and Engineering

A major goal of omics-driven research is the practical application of molecular discoveries to develop stress-resilient cultivars. Strategies include marker-assisted selection (MAS) based on QTLs and SNPs linked to antioxidant capacity, the introgression of stress-inducible antioxidant genes into elite cultivars, and the CRISPR/Cas-mediated editing of promoter or enhancer regions to boost stress-responsive gene expression [107].

In rice, MAS and QTL mapping have been applied to enhance antioxidant capacity and drought tolerance through the incorporation of loci associated with glutathione metabolism and ROS-scavenging enzymes [108]. Genome editing of regulatory regions in *Arabidopsis* has also demonstrated that manipulating promoter architecture can enhance tolerance to oxidative stress by fine-tuning gene expression [109].

Beyond classical gene editing, the engineering of synthetic TFs and synthetic promoters has emerged as a powerful strategy to achieve precise and conditional control of antioxidant gene activation. For example, synthetic zinc finger and TALE (Transcription Activator-Like Effector)-based TFs have been designed to upregulate APX, CAT, and SOD genes under stress-inducible promoters, resulting in improved tolerance to oxidative and abiotic stress without significant growth penalties in *Arabidopsis* and rice [110,111]. Similarly, synthetic promoters constructed by fusing minimal core sequences with ROS- or stress-responsive cis-elements have been shown to drive strong but transient antioxidant gene expression only under stress conditions, thereby minimizing energy expenditure during optimal growth [112].

From a regulatory perspective, plants developed using synthetic TFs or synthetic promoters via stable transformation are generally classified as genetically modified organisms (GMOs) under most current legislative frameworks (e.g., EU Directive 2001/18/EC; USDA-APHIS guidelines). However, plants obtained through transient expression or without foreign DNA integration (e.g., through segregation of the transgene) may be regulated differently depending on the jurisdiction [113].

### 4.8. Species-Specific Antioxidant Genes and Their Biotechnological Relevance in Crops

Cultivated crops exhibit species-specific antioxidant strategies shaped by their physiological roles and genomic architecture. In rice, overexpression of *OsCATC* and *OsAPX1* enhances H_2_O_2_ detoxification and maintains photosynthetic efficiency under salinity stress [114,115]. In wheat, elevated expression of *GSH1* improves ROS buffering and membrane stability during drought, supporting cellular integrity under water-limited conditions.

In tomato, SlWRKY31 modulates both antioxidant enzyme expression and flavonoid biosynthesis, while SlSOD1 contributes to ROS scavenging and secondary metabolite regulation [116,117,118]. Additional studies have confirmed that OsCATC enhances photosynthesis and antioxidant capacity in rice under salt stress, and that *OsAPX1* plays a critical role in maintaining redox homeostasis under stress conditions. The coordinated function of SlWRKY31 in regulating flavonoid metabolism and ROS detoxification highlights its dual role in secondary metabolism and antioxidant defense [119]. These gene-level interventions underscore the importance of tailoring antioxidant engineering strategies to the stress-responsive profiles and redox requirements of individual crops.

Such precision approaches hold promise for optimizing tolerance traits while preserving species-specific physiological balance.

## 5. Biotechnological Strategies and Translational Breeding for Redox-Based Stress Resilience

With the increasing understanding of redox signaling and antioxidant gene networks, biotechnological and breeding innovations are now redefining how crops can be engineered to withstand oxidative stress. This section highlights translational strategies, from classical gene overexpression to synthetic modules and AI-enhanced breeding, that bridge molecular insights and field-level resilience. In addition, the integration of genetic and biotechnological strategies with biostimulant applications, including seaweed extracts, humic substances, protein hydrolysates, and beneficial microorganisms, may offer complementary benefits for enhancing antioxidant capacity and oxidative stress tolerance. Such combined approaches could contribute to more sustainable crop management practices and improved resilience under challenging environmental conditions [120].

### 5.1. Classical Transgenic Approaches: Overexpression of Redox Enzymes

Early biotechnological approaches to improving oxidative stress tolerance in plants focused on the transgenic overexpression of genes encoding antioxidant enzymes such as SOD, APX, CAT, and GPX [121]. These efforts demonstrated significant improvements in tolerance to abiotic stressors including drought, salinity, heavy metals, and high light intensity. Transgenic tomato lines overexpressing SlSOD showed improved tolerance to oxidative stress, highlighting the functional role of SOD in redox homeostasis. Similarly, overexpression of chloroplastic AtAPX1 in *Arabidopsis* conferred enhanced salt tolerance and reduced oxidative damage. In rice, overexpression of *OsCATC* improved H_2_O_2_ detoxification, photosynthetic performance, and biomass accumulation under salinity stress.

Later studies confirmed that simultaneous expression of multiple antioxidant enzymes, such as SOD and APX, improved overall redox balance and stress resilience in transgenic tobacco. Even greater effects were observed in tomato lines expressing stacked APX and GR, where synergistic antioxidant activity led to improved stress memory and faster responses upon re-stimulation [122].

These findings are consistent with metabolomic analyses linking antioxidant gene overexpression to enhanced redox buffering and metabolic adaptation. In maize, overexpression of mitochondrial Mn-SOD significantly improved tolerance to low-temperature stress, indicating the importance of organelle-specific protection. More recently, overexpression of cytosolic APX1 in tobacco was shown to enhance drought tolerance and confer long-term molecular stress memory [123].

### 5.2. Transcription Factor Engineering for Coordinated Antioxidant Control

In addition to structural antioxidant enzymes, modern biotechnological strategies have focused on the manipulation of TFs, which orchestrate the expression of entire ROS-responsive gene networks. This approach enables broader and more dynamic regulation of antioxidant defenses compared to single-gene engineering.

Early work demonstrated that overexpression of ERF1B increased oxidative stress tolerance in *Arabidopsis* through upregulation of redox-related genes [124]. Similarly, DREB2A, another well-characterized TF, was shown to enhance tolerance to drought and heat stress via transcriptional activation of stress-inducible antioxidant enzymes [125]. In rice, OsNAC6 was identified as a critical regulator of abiotic stress responses, contributing to both antioxidant activation and stress signaling [126]. Likewise, WRKY30 overexpression in *Arabidopsis* improved redox homeostasis and disease resistance by enhancing antioxidant enzyme activity. Studies in tomato confirmed the central role of WRKY TFs in coordinating both stress-responsive gene expression and antioxidant defense. In particular, SlWRKY31 has been shown to regulate oxidative stress responses and improve drought tolerance when overexpressed, as well as modulate flavonoid biosynthesis and ROS signaling under salinity stress.

Recent advances include the development of synthetic transcription factor circuits that target entire antioxidant pathways, offering greater precision and conditional control in crops. Moreover, targeted engineering of *OsNAC6* in rice confirmed its ability to activate redox pathways and enhance tolerance to drought and salinity.

### 5.3. Genome Editing of Redox Pathways with CRISPR/Cas

The advent of CRISPR/Cas-based genome editing technologies, particularly CRISPR/Cas9 and Cas12a, has revolutionized plant biotechnology by enabling precise and efficient modifications of antioxidant-related genes [127]. These tools allow edits not only in structural genes (e.g., *APX1*, *GSH1*) but also in regulatory elements such as promoters or enhancers of redox-responsive TFs.

One early application involved the CRISPR/Cas9-mediated knockout of *RBOHD* in tomato, leading to reduced oxidative damage and enhanced drought tolerance [128]. Similarly, promoter editing of *OsAPX1* in rice enabled stress-inducible expression, improving salt tolerance and dynamic antioxidant regulation. Targeted edits using Cas12a to upregulate GSH1 in maize successfully boosted glutathione production and drought resilience. More recently, base editing approaches have been applied to fine-tune the catalytic efficiency of enzymes such as APX1, resulting in improved ROS detoxification under stress conditions. CRISPR has also been used to eliminate excessive ROS production [129]. In chickpea, deletion of *RBOHF* decreased H_2_O_2_ accumulation and improved salt stress resilience. Promoter engineering of *AtSOD1* via CRISPR further demonstrated the potential to modulate expression levels in response to environmental cues, enhancing heat tolerance in *Arabidopsis*. Base editing with Cas12a targeting SlRBOH1 in tomato led to improved fruit oxidative stress resistance [130]. Notably, genome-edited rice lines with modified *OsDi19* exhibited improved drought and salt tolerance without foreign DNA, thus bypassing GMO classification in some countries. CRISPR/Cas9 editing of *OsAPX2* in sugarcane confirmed the utility of redox enzyme targeting in heat stress scenarios, while recent work demonstrated multiplex genome editing of *GSH1* and *GRXS17* in *Arabidopsis,* resulting in enhanced oxidative stress resilience and stable growth plant development [131,132].

### 5.4. Molecular Breeding Empowered by MAS, Multi-Omics, and AI

While genome editing offers precision, molecular breeding has been significantly enhanced by tools such as MAS.

MAS enables breeders to select alleles linked to antioxidant traits using molecular markers such as SNPs and SSRs, allowing the incorporation of redox-resilient traits into elite cultivars. In tomato, wheat, and rice, MAS has identified cultivars with enhanced glutathione metabolism, elevated antioxidant enzyme expression, and improved redox cycling under stress conditions. For instance, QTLs linked to GSH1, MDHAR, and CAT1 have been introgressed into high-yielding varieties, enhancing tolerance to drought, salinity, and oxidative environments [133].

Integrating MAS with transcriptomics and metabolomics enables the parallel tracking of genetic and biochemical markers of oxidative resilience. In maize, breeding lines selected via MAS for high ascorbate content and elevated *ZmSOD4* expression outperformed controls in field trials under UV and nutrient stress.

In recent years, AI and ML have been integrated into breeding pipelines to predict antioxidant gene performance and prioritize genomic targets for selection. ML models that incorporate multi-omics data now assist in predicting gene–trait relationships in redox regulation. For example, the AI-ROS initiative has demonstrated that integrative modeling accelerates the identification of candidate genes linked to redox tolerance traits [134].

In tomato, genome-wide MAS has been combined with AI tools to identify and rank alleles associated with drought and oxidative stress tolerance. These lines, initially pre-selected through MAS, were subsequently refined using CRISPR-based promoter editing, enabling dynamic redox activation in response to drought and heat. Genomic selection supported by AI-ROS prediction has also been successfully applied in rice and wheat to forecast the functional impact of antioxidant-related genes under environmental stress [135,136]. Additionally, enviromics-assisted genomic prediction, which incorporates climate and soil data, has enabled breeders to prioritize redox traits in fluctuating environments such as saline soils, high altitudes, and semi-arid regions [137,138].

### 5.5. Synthetic Biology and Ethical Considerations in Antioxidant Engineering

A frontier in antioxidant engineering involves the application of synthetic biology, which allows the rational design of regulatory modules that are conditionally activated under oxidative stress [139]. Recent advancements include the construction of synthetic promoters that integrate ROS, ABA, and pathogen-responsive elements, enabling fine-tuned expression of antioxidant genes in response to multiple stimuli. Additionally, modular gene circuits have been developed to activate stress responses only when ROS levels exceed specific physiological thresholds, improving stress responsiveness and minimizing metabolic costs [140]. Another promising strategy includes the use of chimeric TFs that couple redox signals with hormone pathways such as ABA, enabling crosstalk-sensitive regulation of gene expression. These synthetic modules enhance the robustness and plasticity of antioxidant networks, particularly under complex or fluctuating environmental conditions. Despite these innovations, important ethical and regulatory challenges remain. Key concerns include public trust, risk perception, and inclusive governance of synthetic biology in agriculture. Furthermore, issues of intellectual property and patent concentration pose barriers to equitable technology dissemination, especially in low- and middle-income countries. On the regulatory front, CRISPR-edited plants that do not contain transgenes are not considered GMOs in several countries, including the United States, Japan, and Argentina, facilitating their release and commercialization [141,142]. Nevertheless, public acceptance remains limited, and transparent, science-based communication is critical to increase societal trust. Ethical issues also arise around food sovereignty, the right of communities to retain control over local seeds and agricultural practices. In this context, the genome editing of local germplasm for competitive traits must be carried out with sensitivity to cultural, ecological, and socioeconomic dynamics. At the molecular level, redox resilience is now viewed as a hub integrating multiple signaling streams. Recent systems biology studies reveal that ROS, reactive nitrogen species (RNS), and reactive sulfur species (RSS) operate synergistically to coordinate transcriptional, post-translational, and metabolic stress networks [143]. This complexity also raises questions regarding field deployment and biosafety, especially when synthetic circuits are released into heterogeneous environments.

**Figure 3:**
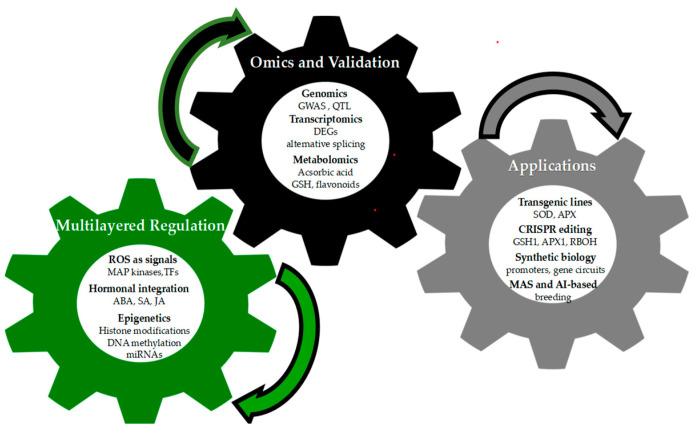
Integrated framework for antioxidant defense in plants. The three interconnected gears represent Multilayered Regulation (ROS signaling, hormonal integration, epigenetics), Omics and Validation (genomics, transcriptomics, metabolomics), and Applications (transgenic lines, CRISPR editing, synthetic biology, MAS/AI-based breeding), highlighting their continuous interaction to enhance oxidative stress tolerance.

To provide a concise visual synthesis of the concepts discussed in Section 3, Section 4 and Section 5, Figure 3 illustrates the integrated framework for antioxidant defense in plants. The diagram highlights the continuous interaction between multilayered regulatory mechanisms, omics platforms combined with functional validation approaches, and applied biotechnological strategies, underscoring their collective role in enhancing oxidative stress tolerance.

## 6. Redox-Driven Innovation: Future Directions and Strategic Challenges

Plant antioxidant research is entering a transformative phase driven by the convergence of computational biology, systems science, and biotechnology. Biotechnological and breeding innovations are now redefining how crops can be engineered to withstand oxidative stress. This section highlights translational strategies, from classical gene overexpression to synthetic modules and AI-enhanced breeding, that bridge molecular insights and field-level resilience. Emerging approaches aim to develop redox-intelligent plant systems capable of dynamically sensing and responding to environmental cues.

### 6.1. From Gene Lists to Redox Networks: The Rise of Systems Biology

Moving beyond the identification of individual genes, systems biology enables researchers to model redox responses as emergent network behaviors. Rather than focusing on isolated components, this approach considers the dynamic interplay of genes, proteins, and metabolites within an integrated regulatory framework.

A major challenge remains understanding how antioxidant genes function collectively and contextually, especially under fluctuating environmental conditions. This challenge is now being addressed through the integration of transcriptomic, proteomic, and metabolomic data to reconstruct ROS-responsive interaction networks. These multi-omics frameworks effectively capture nonlinear behaviors, cross-pathway crosstalk, and context-dependent activation of redox signaling modules [144].

For example, co-expression and network modeling studies in tomato have revealed functional bZIP–NPR1 modules that serve as central integrators of hormone and redox pathways. Similarly, multi-omics studies in *Zea mays* and *Populus* have identified RBOH-centered modules that respond to salt and oxidative stress [145,146].

Advances in time-resolved omics now allow researchers to monitor the dynamic evolution of ROS signaling networks. In *Arabidopsis*, the integration of proteomics and metabolomics has been used to model changes in redox nodes during stress adaptation [147]. Computational methods such as machine learning and topology-based analysis have also identified key hub regulators, genes or proteins whose perturbation disproportionately impacts overall system behavior [148].

Computational methods such as machine learning and topology-based analysis have also identified key hub regulators, genes or proteins whose perturbation disproportionately impacts overall system behavior.

These regulatory hierarchies and network motifs provide a conceptual blueprint for engineering redox resilience. In particular, feedback modules involving NPR1, bZIP transcription factors, and RBOH enzymes govern trade-offs between growth and defense. Such systems-level insights are critical for rational intervention and for designing crops capable of flexibly responding to oxidative challenges [149,150,151].

### 6.2. Artificial Intelligence and Predictive Redox Biology

AI, particularly ML and deep learning (DL), is revolutionizing how plant scientists explore multi-omics datasets. AI models can now classify stress-responsive gene expression profiles, uncover hidden regulatory motifs, and predict cis-elements in redox-inducible promoters [152]. Through automated pipelines, thousands of genes can be prioritized based on network centrality, transcriptional dynamics, and environmental responsiveness, expediting the identification of redox-regulated modules. Simultaneously, AI technologies are used to integrate genomic data (e.g., SNPs), phenotypic traits (e.g., chlorophyll fluorescence), and environmental parameters, accelerating the screening of oxidative stress tolerance [153,154,155]. Synthetic promoter design has also been transformed; DL models can now simulate optimal transcription factor binding site configurations to enhance redox sensitivity. In *Nicotiana*, AI-designed synthetic promoters have demonstrated strong ROS inducibility with minimal fitness penalties. Recent advances include modeling of redox circuits using programmable logic gate behavior, enabling precise control of antioxidant responses under defined redox thresholds. Machine learning approaches integrating multi-omics datasets assist in prioritizing key antioxidant genes by ranking them according to network topology and transcriptional response, while predictive genotype-to-phenotype models have been developed to forecast redox resilience in wheat [156,157]. Finally, regulatory circuits exhibiting logic behavior, designed with deep learning networks, have been validated in *Arabidopsis* and *Nicotiana*, demonstrating the feasibility of AI-guided programmable architectures. AI-driven multi-omics integration has further enabled the identification of dynamic and responsive redox networks under fluctuating environmental conditions [158].

### 6.3. Synthetic Biology for Modular Redox Engineering

Synthetic biology offers programmable solutions for managing oxidative stress in plants by enabling precise spatiotemporal control of antioxidant responses. One notable innovation is the construction of synthetic gene circuits that activate redox defenses only when ROS exceed defined thresholds, thereby improving stress responsiveness while minimizing energy costs. A complementary strategy involves the development of chimeric transcription factors (synTFs) that couple redox regulation with hormonal signaling [159]. For example, TFs combining NAC DNA-binding domains with MYB activation domains have been shown to enable dual control over stress-inducible gene expression in tobacco [160]. To avoid crosstalk with native gene networks, researchers have incorporated orthogonal elements, regulatory sequences and TFs derived from fungi, bacteria, or viruses, which respond to plant-like cues but function independently from endogenous pathways. Fungal promoter–TF modules, for instance, have been adapted for precise ROS responsiveness in synthetic designs in fungi [161]. In plants, TPP riboswitches are well-documented regulatory elements in species such as *Arabidopsis* and *Oryza sativa* [162,163], and bacterial riboswitches could, in principle, be engineered as modular redox sensors in crops, although no plant-specific applications have yet been reported [164].

**Figure 4:**
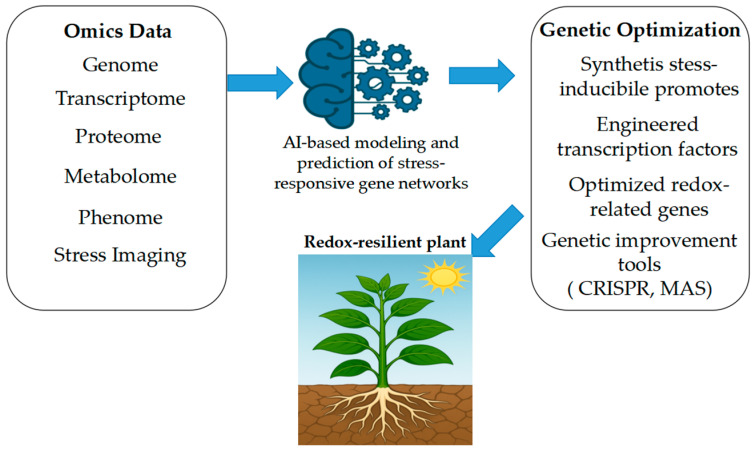
Integration of omics sciences and AI for plant genetic optimization under stress conditions. Multi-omics platforms (genomics, transcriptomics, proteomics, and metabolomics) generate high-dimensional datasets that, when analyzed using AI and machine learning algorithms, support the identification of key genes, regulatory networks, and stress-responsive traits. This integrated approach enables targeted breeding and genetic optimization of plants for improved resilience to environmental stressors.

Logic-based control systems are also gaining traction. Synthetic promoters engineered with AND logic gates have been used to drive expression of *APX* under dual-stress signals, ensuring activation only in specific environmental contexts. Similarly, dual-domain synTFs that integrate bZIP and ERF elements provide layered regulation in maize, coordinating antioxidant and hormonal signaling pathways. Recent synthetic biology studies have proposed the adaptation of toggle switches built from bacterial-origin sensor–repressor modules for reversible redox-dependent control of gene expression in *Arabidopsis*, although their stable implementation in plants has yet to be demonstrated experimentally [165]. Synthetic promoter libraries screened in tomato have enabled selection of constructs that are highly inducible by oxidative stress but impose minimal metabolic load [111,166]. Further refinements include the use of orthogonal viral enhancer elements, which enable ROS-specific expression without disrupting native promoter architecture [167]. Overall, the field is moving toward modular and predictable design, allowing synthetic circuits to function reliably across species and environmental contexts (Figure 4).

### 6.4. Limitations and Future Challenges

Despite significant advances, limitations and future challenges remain in redox-based plant biotechnology.

First, overexpression of antioxidant enzymes frequently imposes metabolic and fitness costs, especially under non-stress conditions, leading to reduced yield potential. Furthermore, gene-by-environment (G × E) interactions often limit field performance—traits engineered for oxidative stress in controlled environments may not translate effectively to field settings.

In genome editing, persistent challenges include off-target effects, low editing efficiency, and limited multiplexing capacity when attempting to alter multiple redox genes simultaneously [168]. Additionally, reliable delivery of CRISPR/Cas systems remains a bottleneck in many crop species. On the regulatory front, inconsistent frameworks across regions hinder the deployment of edited crops, with countries diverging on definitions, safety assessments, and trade barriers. Technically, integrating multi-omics datasets for breeding remains computationally demanding, requiring advanced algorithms and infrastructure to handle large-scale data effectively. Non-technical challenges also loom large. Public perception of gene-edited crops remains cautious, and transparent risk communication is essential to gain trust and acceptance. Moreover, intellectual property constraints and the need to preserve germplasm sovereignty, especially in lower-income countries, pose significant barriers to equitable technology access [169,170].

### 6.5. Redox-Informed Strategies for Climate Resilience and Ethical Innovation

As climate change intensifies, combinatorial abiotic stressors, such as drought, salinity, heat, and ozone, amplify oxidative pressure in plants, positioning redox regulation as a pivotal determinant of adaptive resilience. Recent research highlights the integration of antioxidant pathways with circadian rhythms and localized ROS networks as effective strategies to enhance cross-tolerance and stress memory, as demonstrated in rice, tomato, and poplar. Epigenetic modifications, including histone marks (e.g., H3K4me3) and DNA methylation, are increasingly recognized as mechanisms for encoding redox-related stress memory and developmental plasticity [171,172].

Advances in omics, AI, and synthetic biology are now converging to enable the development of redox-intelligent crops—engineered systems that dynamically sense and respond to oxidative cues through synthetic sensor–actuator circuits. For instance, programmable miRNA–TF modules in rice and tomato have been shown to modulate antioxidant activity in real time, improving resilience with minimal energy cost. AI-assisted platforms further optimize promoter logic and gene circuit behavior, paving the way for precision agriculture under fluctuating environmental conditions [156,157,173].

However, these technological advances raise critical ethical and governance considerations, including data transparency, equity in access, ecological risk, and community consent—particularly in vulnerable regions. As synthetic redox networks become more complex, ensuring their metabolic stability, biosafety, and scalability is essential. Ultimately, integrating redox biology with responsible innovation frameworks offers a path toward resilient, sustainable, and ethically grounded crop systems [174,175].

## 7. Conclusions

Oxidative stress remains a pervasive limitation on plant productivity, particularly given the compound challenges of climate change. This review has highlighted how plant antioxidant systems act as dynamic interfaces that translate environmental signals into physiological responses, coordinated through multilayered genetic, epigenetic, and biochemical regulation. The integration of omics platforms, synthetic biology, and AI is reshaping our ability to understand, model, and reprogram redox networks. Far from being static defense systems, antioxidants are emerging as central regulators of plant resilience that are tightly connected to developmental timing, signaling plasticity, and environmental sensing. Through gene editing, synthetic circuits, and multi-omics breeding, antioxidant pathways can now be strategically optimized, rather than passively inherited. Future efforts must prioritize interdisciplinary innovation, combining high-throughput data, computational modeling, and translational field applications, to deliver next-generation crops that are resilient, efficient, and adaptable. Redox-intelligent agriculture thus emerges as a promising paradigm to improve crop performance, foster ecological sustainability, and safeguard food sovereignty in an era of escalating environmental challenges.

## Figures and Tables

**Table 1:** Overview of key ROS, their sources, stress-related roles, and functions in plants.

ROS	Full Name	Main Source	Abiotic Stress	Biotic Stress	Function
^1^O_2_	Singlet Oxygen	Chloroplasts (PSII, chlorophyll excitation)	High light, drought, cold	Indirectly via signaling during infection	Highly reactive and signaling [8,9]
O_2_^−^•	Superoxide Anion	Mitochondria, chloroplasts, NADPH oxidases	Cold, salinity, heavy metals	Pathogen-induced oxidative burst	ROS precursor, signaling [2,5]
H_2_O_2_	Hydrogen Peroxide	SOD activity, oxidases, photosystems	All abiotic stresses	Elicitor-induced signaling	Central signaling molecule [5,6,12]
•OH	Hydroxyl Radical	Fenton reaction (Fe^2+^ + H_2_O_2_)	UV, heavy metals	Localized cell death (hypersensitive response)	Extremely toxic, causes damage [28]
